# Travel time to cataract surgical services in Kenya, Malawi and Rwanda: demonstrating a standardised indicator of physical access to cataract surgery

**DOI:** 10.1038/s41433-023-02790-8

**Published:** 2023-10-18

**Authors:** Ian McCormick, John M. Nesemann, Jinfeng Zhao, Shaffi Mdala, Gatera Fiston Kitema, Nyawira Mwangi, Michael Gichangi, Kevin Tang, Matthew J. Burton, Jacqueline Ramke

**Affiliations:** 1 International Centre for Eye Health, London School of Hygiene & Tropical Medicine, London, UK; 2 University of California San Francisco, Department of Ophthalmology, San Francisco, CA USA; 3 School of Population Health, University of Auckland, Auckland, New Zealand; 4 Kamuzu University of Health Sciences, Blantyre, Malawi; 5 Queen Elizabeth Central Hospital, Blantyre, Malawi; 6 Ophthalmology Department, School of Health Sciences, University of Rwanda, Kigali, Rwanda; 7 Kenya Medical Training College, Nairobi, Kenya; 8 Ophthalmic Services Unit, Kenya Ministry of Health, Nairobi, Kenya; 9 Department of Population Health, London School of Hygiene & Tropical Medicine, London, UK; 10 National Institute for Health Research Biomedical Research Centre for Ophthalmology at Moorfields Eye Hospital NHS Foundation Trust and UCL Institute of Ophthalmology, London, UK; 11 School of Optometry and Vision Science, University of Auckland, Auckland, New Zealand

**Keywords:** Public health, Lens diseases

## Abstract

**Background:**

Travel time can be used to assess health services accessibility by reflecting the proximity of services to the people they serve. We aimed to demonstrate an indicator of physical access to cataract surgery and identify subnational locations where people were more at risk of not accessing cataract surgery.

**Methods:**

We used an open-access inventory of public health facilities plus key informants in Kenya, Malawi and Rwanda to compile a geocoded inventory of cataract facilities. For each country, gridded estimates of the population aged ≥ 50 years and a travel-time friction surface were combined and a least-cost-path algorithm applied to estimate the shortest travel time between each grid and the nearest cataract facility. We categorised continuous travel time by 1-, 2- and 3 h thresholds and calculated the proportion of the population in each category.

**Results:**

At the national level, the proportion of the population aged ≥ 50 years within 2 h travel time to permanent cataract surgical services was 97.2% in Rwanda (*n* = 10 facilities), 93.5% in Kenya (*n* = 74 facilities) and 92.0% in Malawi (*n* = 6 facilities); this reduced to 77.5%, 84.1% and 52.4% within 1 h, respectively. The least densely populated subnational regions had the poorest access to cataract facilities in Malawi (0.0%) and Kenya (1.9%).

**Conclusion:**

We demonstrated an indicator of access that reflects the distribution of the population at risk of age-related cataract and identifies regions that could benefit from more accessible services. This indicator provides additional demand-side context for eye health planning and supports WHO’s goal of advancing integrated people-centred eye care.

## Introduction

In the context of universal health coverage, eye health planners require information to address inequities in service provision and achieve universal eye health. Data on eye health conditions and outcomes can be disaggregated by key equity dimensions, including gender, socioeconomic position and geographic location, to identify and address gaps in service provision to ensure no one is left behind.

Cataract is the leading cause of blindness worldwide, with age-standardised prevalence highest in the South Asia and sub-Saharan Africa regions [1]. Cataract primarily affects older people and if untreated, is associated with reduced quality of life and worse socioeconomic position [2].

The accessibility of eye care services can be considered in terms of their availability, affordability and acceptability but also geographically, in terms of the population’s proximity to care, measured in minutes of travel time [3, 4]. Travel time as a measure of access to emergency surgery has received considerable attention in the literature [5, 6], but travel time to other types of surgical care—including for cataract—will also likely influence whether or not a patient accesses it [7].

People living in rural or remote locations are at risk of poorer access to cataract services [8–10]. For rural populations in low-income settings these challenges are likely to be exacerbated by additional barriers related to transport, infrastructure and cost of travel [4, 11]. In response, in the inaugural *World Report on Vision*, the World Health Organization (WHO) highlighted the need to strengthen eye care service delivery in rural areas, alongside the need to improve data to guide service planning for remote communities [12].

Indicators of health care accessibility have historically included health worker and health facility density and distribution per capita [13, 14]; this approach to monitoring the coverage of eye care service delivery by subnational health administrative areas was proposed as an eye health indicator as long ago as 2002 [15]. More recently, geocoded inventories of public health facilities have been made available and indicators of the travel time required to access emergency surgery and maternal and newborn health care have been developed and used to quantify the proportion of populations residing beyond a desired threshold of travel time [5, 7, 16]. While these models have provided useful insights about the geographic accessibility of other health services, this approach has not yet been extended to characterise access to eye care services.

The geographical coordinates of cataract surgical facilities relative to the spatial distribution of the population 50 years and older has not previously been analysed. In this study we aimed to demonstrate an indicator of physical access to cataract surgery, to identify subnational locations where people were more at risk of not accessing cataract surgery. We do this using data on population density and eye care facility locations in a cost distance analysis, using longer travel time as a proxy for being at greater risk of not accessing cataract surgical services.

## Methods

### Ethics

Ethical approval for the study was granted by the London School of Hygiene & Tropical Medicine Research Ethics Committee (Ref 17875).

### Cataract facility data

We used an open-access inventory of 96,395 public health facilities (including those run by government, faith-based and non-governmental organisations) in sub-Saharan Africa (50 countries) [17] as the basis for an audit of facilities providing cataract services in three countries: Kenya, Malawi and Rwanda. A ‘key informant’ for the eye health sector in each country (NW & MG, Kenya; SM, Malawi; GFK, Rwanda) was identified through existing network of contacts within the International Centre for Eye Health. These key informants reviewed a country-specific subset of facilities from the inventory described above and added additional information about cataract services for each one listed. Key informants most recently reviewed the lists for completeness in February 2023. Each country list was filtered to exclude the lowest level facility type (dispensaries or health posts) where we presumed cataract surgery would never be performed. Key informants identified facilities offering cataract surgical services on either a permanent or occasional basis, or never, according to the options shown in Table 1. Key informants added the details of any non-private sector cataract surgery facilities not included in the original dataset.

**Table 1:** Levels of cataract surgical service identified per facility.

Cataract surgical service status	Definitions
Permanent	Ophthalmic staff based at the facility performing cataract surgery all year (except in extraordinary circumstances)
Occasional	Ophthalmic staff performing cataract surgery not permanently based at the facility, surgery offered on an ad hoc basis
None	Cataract surgery is never performed at this facility

### Proposed indicator

The proposed indicator is *the proportion of the population aged 50 years and older able to access any non-private sector health facility providing permanent cataract surgical services within a given travel time*; reported nationally and at the most appropriate subnational administration level for a given country. To demonstrate, we did calculations at 1, 2, and 3 h travel-time thresholds nationally and reported at a 2 h travel-time threshold for subnational physical access to permanent cataract facilities. We did not include facilities with only occasional service provision because, in a recent study, establishing permanent surgical capacity at a district level was the most highly ranked strategy to improve access to cataract surgery globally, and for sub-Saharan Africa [10]. Additionally, in the sub-Saharan Africa region, a retrospective study indicated the presence of a permanent cataract facility in a location may be associated with improved cataract surgical coverage [18]. We found no previously proposed thresholds for travel time as a barrier to accessing cataract services in the literature. We used a 2 h travel-time threshold for subnational values based on its use in studies of access to other surgical services and in conjunction with feedback from our key informants [5–7].

### Population data

We obtained estimates of the population 50 years and older in Kenya, Malawi and Rwanda from WorldPop (https://www.worldpop.org/) at 100 m^2^ spatial resolution [19]. Top-down constrained population estimates (i.e., estimates of population density constrained to areas containing built settlements) for 2020 were downloaded and 5-year age-sex group counts were aggregated for men and women aged 50 years and older. We used constrained estimates to avoid non-zero population numbers in uninhabited areas because accurate estimation of population density is important for studies of accessibility [20]. We aggregated population estimates to 1 km^2^ resolution to align with the spatial resolution of the friction surface raster and to make population density patterns more visible in part A of each country Figure; all population percentages calculated for bar chart legends and the comparison of facility density values with our indicator output at the provincial level in Rwanda used the original 100m^2^ population resolution for greater accuracy.

### Physical access modelling – a cost distance analysis

An open-access friction surface was downloaded from the Malaria Atlas Project via the malariaAtlas package in R [21]. The methodology behind the construction of the friction surface has been described in depth elsewhere [22, 23], but briefly the authors created a map of the world at a 1 km^2^ resolution, using transportation data from Google and Open Street Maps; each 1 km^2^ pixel was then assigned a travel time required to cross that pixel defined using the modes of transport available in that pixel (e.g., roads of varying speeds, navigable bodies of water, railways, or walkable terrain and the slope of that terrain), with the fastest mode of transport taking priority. After cropping the friction surface to the countries of interest, the point locations of each permanent cataract surgery facility were converted to a matrix of latitude and longitude coordinates and plotted on the friction surface. A least-cost-path algorithm was then applied to the friction surface to calculate pixel-level travel time for the optimal (i.e., shortest travel time) path between each pixel and the nearest cataract facility, assuming motorised transport was always used where available [24].

### Analysis

Continuous travel time for each 1 km^2^ unit was categorised as (1) one hour or less, (2) more than one and less than or equal to two hours, (3) more than two and less than or equal to three hours, and (4) more than three hours travel from the nearest facility. We overlayed population data for each country on the categorised travel time rasters and calculated the proportion of individuals aged 50 years and older in each travel time category. All analyses were done using R software (version 4.2.2) [25].

### Comparison of cost distance analysis with provider-to-population ratio values in Rwanda

In addition to using district level (admin level 2) units for the indicator in Rwanda, we used admin level 1 provinces to compare our indicator with a provider-to-population ratio, i.e., the number of permanent cataract facilities per 100,000 people aged 50 years and older per province [24, 26]. We used this higher admin level as not all districts had a permanent facility for comparison.

## Results

**Fig. 1:**
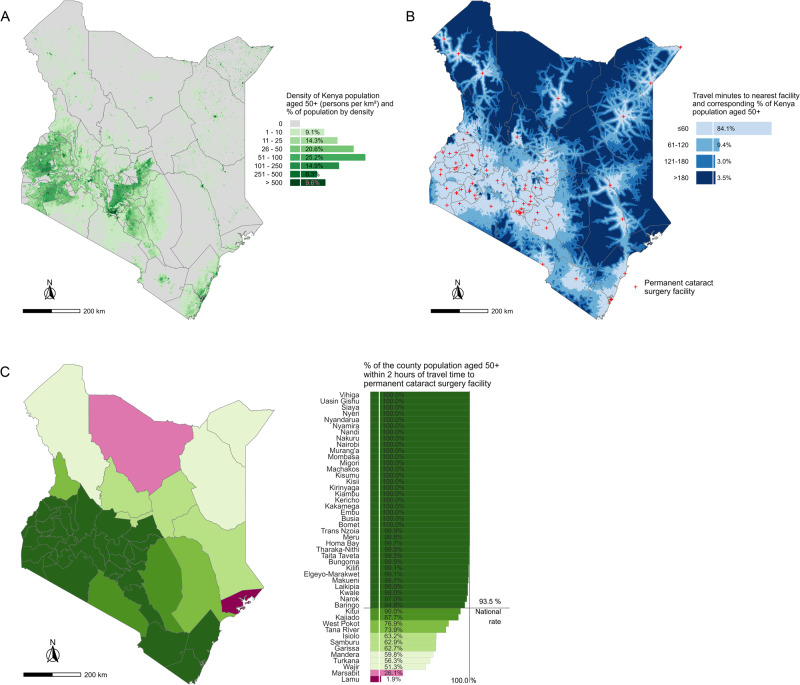
Physical access to cataract surgery for the population aged ≥50 years in Kenya. **A** Distribution and density of population aged ≥ 50 years in Kenya. **B** Proportion of population aged ≥ 50 years in Kenya by category of travel time to the nearest permanent cataract surgical facility. **C** The proportion of each Kenyan county’s population aged ≥ 50 years living within 2 h travel time to the nearest permanent cataract surgical facility.

**Fig. 2:**
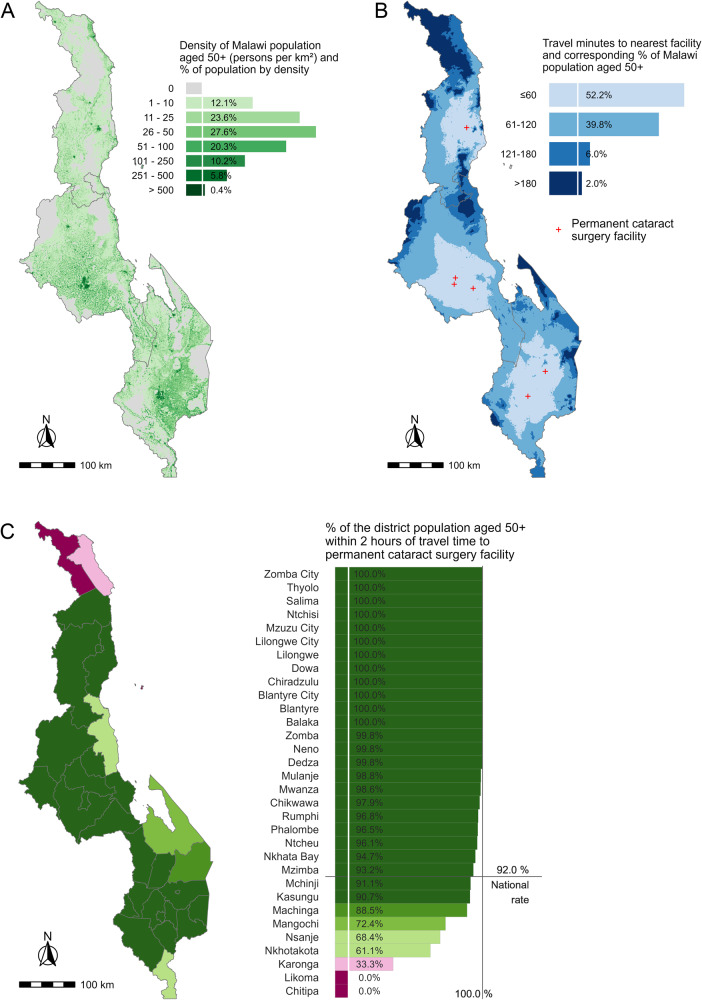
Physical access to cataract surgery for the population aged ≥50 years in Malawi. **A** Distribution and density of population aged ≥ 50 years in Malawi. **B** Proportion of population aged ≥ 50 years in Malawi by category of travel time to the nearest permanent cataract surgical facility. **C** The proportion of each Malawian district’s population aged ≥ 50 years living within 2 h travel time to the nearest permanent cataract surgical facility.

**Fig. 3:**
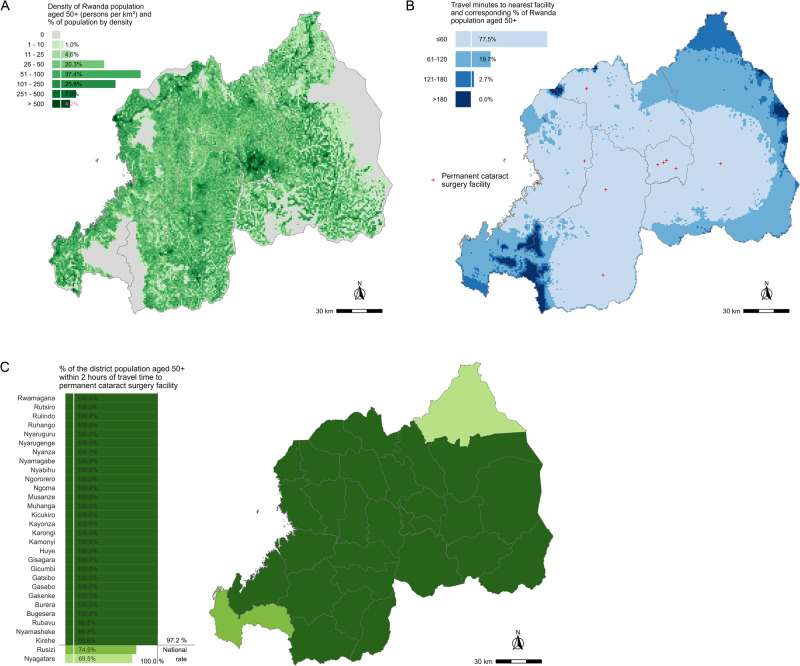
Physical access to cataract surgery for the population aged ≥50 years in Rwanda. **A** Distribution and density of population aged ≥ 50 years in Rwanda. **B** Proportion of population aged ≥ 50 years in Rwanda by category of travel time to the nearest permanent cataract surgical facility. **C** The proportion of each Rwandan district’s population aged ≥ 50 years living within 2 h travel time to the nearest permanent cataract surgical facility.

Excluding health posts and dispensaries, the master list had 1794 public health facilities in Kenya, 561 in Malawi and 533 in Rwanda. We identified 66, 5 and 9 permanent cataract facilities included in the master list and added 8, 1 and 1 additional permanent facilities for Kenya, Malawi and Rwanda respectively (Figs. 1–3B).

At the national level, the proportion of the population aged 50 years and older living within two hours travel time to cataract surgical services was 97.2% in Rwanda, 93.5% in Kenya and 92.0% in Malawi (Figs. 1–3B). Across the three countries, there was more variation in the proportions living within 1 h travel time of a permanent cataract facility. In Kenya, 84.1% of the population aged 50 years and older were within 1 h compared to 77.5% in Rwanda and 52.4% in Malawi (Figs. 1–3B). In all three countries, only a small percentage of people aged 50 years and older lived more than 2 h from a permanent facility (Kenya 6.5%; Malawi 8.0%; Rwanda 2.7%).

The results for the indicator disaggregated to the most appropriate administrative level are show in Figs. 1–3C, being district level in Malawi and Rwanda and county level in Kenya. In Kenya, counties in the more densely populated centre, west and parts of the south of the country had 100% or close to 100% of their population 50 years and older within 2 h travel time to a permanent facility. Counties in the less densely populated north and east of the country had poorer physical access to cataract services (Fig. 1C). In Malawi, no one aged 50 years or older in the most northern two counties in the North Region lived within 2 h travel time to a permanent facility, whereas the more densely populated, Central and South Regions had much better physical access (Fig. 2C). In Rwanda, there was more homogeneity in physical access by district; only two districts in the extreme north and south-west of the country did not have 100% of the population 50 years and older within 2 h travel time to a permanent facility (Fig. 3C).

### A comparison of provincial-level provider-to-population ratios and population physical access in Rwanda

Rwanda’s 30 districts are distributed across five administrative ‘level 1’ regions – four provinces and Kigali City. Permanent cataract surgical facility density per 100,000 people 50 aged years and older was higher in Western Province than in Southern Province (0.70 vs 0.56 per 100,000) (Table 2). By this metric, physical accessibility of services was better in Western Province than Southern Province. However, if we consider the distribution of the population 50 years and older and travel time to facilities in each province, we reach the opposite conclusion; almost all (97.5%) people aged 50 years and older in Southern Province resided within 1 h travel time of a permanent facility, whereas fewer than two thirds (60.8%) of people aged 50 years and older in Western Province could access permanent cataract services within the same 1 h travel time.

**Table 2:** Comparison of number of facilities, facility density and the proportion of people aged 50 years and older within 1 h travel time to a permanent cataract facility, per province in Rwanda.

Administrative zone	Population 50+ in 2020	*N* permanent facilities	Facility density per 100,000 people 50 +	≤ 1 h travel time to permanent facility
Kigali City	120,117	4	3.33	120,117 (100.0%)
Western Province	284,127	2	0.70	172,693 (60.8%)
Southern Province	358,575	2	0.56	349,658 (97.5%)
Northern Province	203,718	1	0.49	176,206 (86.5%)
Eastern Province	392,710	1	0.25	234,892 (59.8%)

## Discussion

We developed a geocoded inventory of cataract surgical facilities in Kenya, Malawi and Rwanda and demonstrated a new indicator of physical access to cataract surgery among the population aged 50 years and older.

We integrated cataract facility data with gridded population estimates for people aged 50 years and older in each country to produce a population-based indicator of physical access to cataract services. At the national level, physical access to cataract surgery in our three pilot countries was good, with over 90% of the population 50 years and older within 2 h travel time of a permanent facility in all three settings. These findings are comparable with a study of access to emergency care in sub-Saharan Africa which found 100% of the all-age population of Rwanda resided within 2 h of a regional or district hospital, with Malawi at 99% and Kenya at 98% for the same indicator [7]. Our findings also demonstrate how population access to cataract services can vary within countries. In Malawi and Kenya, one or two low population density districts or counties were considerable outliers, with zero or close to zero percent of people aged 50 years and older living within 2 h travel time to a permanent facility. These areas fell far below the national rates. Inclusion of countries with less well-developed health, eye care or road network infrastructure would have demonstrated even more subnational variation. Rwanda is a geographically much smaller country with higher average population density than either Kenya or Malawi; here, there was much more consistency in the indicator value by district, with no district achieving less than 70% of the population aged 50 years and older within 2 h of permanent services.

We found that a 2019 open access list of public health facilities in sub-Saharan Africa provided a suitable basis for a key informant to develop a cataract facility list in each country. While the baseline facility data we used in the study is limited in geographic coverage to one world region, there has recently been considerable emphasis placed on the value of high-quality government-owned Health Facility Master Lists (HFML). For example, WHO has launched a Geolocated Health Facilities Data initiative to support governments to develop accurate, actively maintained and publicly available HFML and to integrate them at a global level [27]. We anticipate that high quality baseline data for building eye care specific geocoded inventories will become more readily available in the coming years, in all world regions. Our key informants were able to supplement the lists of non-private cataract surgery providers with information on fully private and other non-governmental facilities, although we did not include them in this analysis. In many settings, eye care has a history of non-governmental, vertical programme delivery and–depending on the distribution of cataract service provision by sector–these additional data could be added to facility lists.

Previously, the density and distribution of eye care facilities has been prioritised as an indicator of access to eye care, reported as a provider-to-population ratio, e.g., hospitals per million population [14]. Nationally, or subnationally, cataract facility density per administrative area does not tell us anything about their location relative to the distribution of the population (potentially) in need of cataract services. Our proposed indicator of geographic access is population-based and reflects the distribution of the population at risk of age-related cataract; it can provide additional demand-side context for eye health planning and is well aligned with WHO’s goal of promoting and monitoring integrated people-centred eye care. Further, a comparison of provider-to-population ratios across lower-level subnational administration units will return zero values for all districts without a surgical facility. In contrast, the indicator proposed here provides a value for any level of administrative unit of interest to service planners.

We demonstrated how the new indicator can enhance understanding of physical access to cataract services by comparing its output with subnational provider-to-population ratio values for each province in Rwanda. Eastern Province had the lowest facility density per 100,000 people aged 50 years and older and the lowest indicator value for the same population residing within 1 h travel time to a permanent cataract service facility. Considering facility density only, the Northern and Southern Provinces were the next worst-served regions. However, taking into consideration our analysis in each province, there is an indication that improving physical access to permanent cataract surgical services may be better served by prioritising infrastructure development in an appropriate location relative to the population in Western Province before either of Northern or Southern Provinces. We recognise that additional health systems data on cataract services–not considered in this study–would be necessary to inform such planning decisions.

There are limitations to this analysis. Physical accessibility is only one component of access to care and does not imply that cataract services at the included facilities are sufficient to cover the demand, or that they are used [16]. Our proposed indicator should be considered alongside other indicators of cataract service accessibility when planning and evaluating eye care service delivery. Following this study, we anticipate further research can address some limitations and strengthen a standardised methodology for estimating and reporting the indicator.

The first way the approach can be strengthened is through continuous improvement of data inputs, namely eye care facility classification and coordinates from HFML and gridded population data, which themselves rely on quality census data. Where feasible, two key informants could complete the cataract facility listing independently and review the results for any discrepancies. This would be particularly useful in countries with many cataract facilities and active public, private and non-governmental providers. In parallel with improving health facility geodata, there is increasing use of open-access gridded population estimates in health research, policy, and planning [28]. Other gridded population estimates are available besides the WorldPop top-down constrained datasets we used, and the choice of methodology can influence the output [20, 29].

Secondly, a comparison of modelled travel times against values calculated using other open-source tools such as AccessMod (https://www.accessmod.org/) and against patient-reported travel times and modalities. The most realistic estimates of physical access to health care services require context-specific knowledge of health-seeking behaviour and transport options to be built into models [20], which was beyond the scope of this study. We used an open access travel time surface with a resolution of 1 km^2^ and aggregated the gridded population estimates to align with this resolution; however, finer resolution surfaces are possible and may give more accurate travel time estimates where the intention is to inform policy and planning decisions [20]. For elective, non-emergency care such as cataract surgery, patients may choose more affordable travel options, such as a combination of walking and public transport, over the fastest motorised transport option available, as used here. A previous study in Rwanda found patient-reported travel time to emergency obstetric care was 50% longer than modelled estimates [30]; we may be similarly underestimating the journey duration for some patients. We limited analysis to one country at a time – this does not account for possible utilisation of closer cataract facilities in neighbouring countries, where available. Many low- and middle-income settings with a large unmet need for cataract surgery are undergoing increasing urbanisation. This secular trend may naturally reduce the number of people living beyond travel time thresholds in subnational administrative areas. For urban environments, a modified analysis approach may be needed to represent physical access more accurately. The effect of traffic on travel time in large cities (including Nairobi in Kenya) can be considerable and can vary according to the time of day [31]. Google has recently collaborated with maternal health researchers in Nigeria–using the technology that powers Google Maps–to provide more realistic travel time estimates according to these factors [32].

Thirdly, consultation on the most appropriate travel time threshold(s) for within- and between-country comparisons. A travel time threshold of 2 h has been defined for access to essential surgical care, based on the risk of death from childbirth complications for journey times longer than this [6]. For cataract surgery, we found no evidence of travel time thresholds described elsewhere and countries could report the indicator against one or more threshold, in the same way as effective cataract surgical coverage can be reported at more than one cataract surgical threshold [33]. In settings where cataract surgery often involves a planned overnight stay in hospital, a 2 h threshold may be artificially low; however, given many subnational areas in all three countries already had 100% coverage for 2 h’ travel time, we did not increase the threshold further. Travel times based on walking speeds only can also be generated and can highlight the extent of the access challenge faced by those with fewest resources who are unable to afford motorised transport options.

Finally, supplementary analyses integrating these population and travel time data with population-based estimates of eye health outcomes (such as eCSC) and facility-level output data (such as number of surgeries, waiting time for surgery) could demonstrate additional value for eye care service planning. For example, scaling up analysis can model the effect of different service delivery development options on people’s physical access to cataract surgery [16].

We have demonstrated a population-based indicator of physical access that reflects the distribution of the population at risk of age-related cataract. By applying it in three countries, we identified rural subregions with limited physical access to services. Alongside facility data in a country, this indicator can provide additional demand-side context for eye health planning and supports WHO’s goal of advancing integrated people-centred eye care.

## Summary

### What was known before

- People living in rural and remote areas in all world regions have been identified as being at more risk of not accessing cataract surgery. Geocoded inventories of health facilities have been used to estimate the proportion of populations residing beyond a desired threshold of travel time as a measure of access health to care. This has not previously been used to assess access to eye care services.

### What this study adds

- We demonstrated an indicator of physical access to cataract surgery using travel time and identified subnational locations where people were more at risk of not accessing cataract surgery. In contrast to a provider-to-population ratio, the indicator reflects the distribution of the population at risk of age-related cataract relative to available services and provides additional demand-side context for eye health planning.

## Data Availability

The R code and facility locations datasets used for the analysis are available at https://github.com/Jinfeng-Zhao/access-eye-facility-mapping.
